# Novel Multiplexed Plasma Biomarker Panel Has Diagnostic and Prognostic Potential in Children With Hypertrophic Cardiomyopathy

**DOI:** 10.1161/CIRCGEN.123.004448

**Published:** 2024-06-07

**Authors:** Gabriella Captur, Ivan Doykov, Sheng-Chia Chung, Ella Field, Annabelle Barnes, Enpei Zhang, Imogen Heenan, Gabrielle Norrish, James C. Moon, Perry M. Elliott, Wendy E. Heywood, Kevin Mills, Juan Pablo Kaski

**Affiliations:** UCL MRC Unit for Lifelong Health & Ageing, UCL, London, United Kingdom (G.C.).; UCL Institute of Cardiovascular Science, UCL, London, United Kingdom (G.C., J.C.M., P.M.E.).; The Royal Free Hospital, Centre for Inherited Heart Muscle Conditions, Cardiology Department, UCL, London, United Kingdom (G.C.).; Translational Mass Spectrometry Research Group, UCL Institute of Child Health, London, United Kingdom (I.D., E.Z., W.E.H., K.M.).; UCL Institute of Health Informatics Research, Division of Infection and Immunity, London, United Kingdom (S.-C.C.).; Centre for Paediatric Inherited & Rare Cardiovascular Disease, Institute of Cardiovascular Science, London, United Kingdom (E.F., A.B., I.H., G.N., J.P.K.).; Centre for Inherited Cardiovascular Diseases, Great Ormond Street Hospital, London, United Kingdom (E.F., A.B., I.H., G.N., J.P.K.).; UCL Medical School, University College London, London, United Kingdom (E.Z.).; Barts Heart Centre, the Cardiovascular Magnetic Resonance Unit, London, United Kingdom (J.C.M.).; Barts Heart Centre, the Inherited Cardiovascular Diseases Unit, St Bartholomew’s Hospital, London, United Kingdom (P.M.E.).

**Keywords:** biomarkers, cardiomyopathy, hypertrophic, machine learning, death, sudden, cardiac, proteomics

## Abstract

**BACKGROUND:**

Hypertrophic cardiomyopathy (HCM) is defined clinically by pathological left ventricular hypertrophy. We have previously developed a plasma proteomics biomarker panel that correlates with clinical markers of disease severity and sudden cardiac death risk in adult patients with HCM. The aim of this study was to investigate the utility of adult biomarkers and perform new discoveries in proteomics for childhood-onset HCM.

**METHODS:**

Fifty-nine protein biomarkers were identified from an exploratory plasma proteomics screen in children with HCM and augmented into our existing multiplexed targeted liquid chromatography-tandem/mass spectrometry-based assay. The association of these biomarkers with clinical phenotypes and outcomes was prospectively tested in plasma collected from 148 children with HCM and 50 healthy controls. Machine learning techniques were used to develop novel pediatric plasma proteomic biomarker panels.

**RESULTS:**

Four previously identified adult HCM markers (aldolase fructose-bisphosphate A, complement C3a, talin-1, and thrombospondin 1) and 3 new markers (glycogen phosphorylase B, lipoprotein a and profilin 1) were elevated in pediatric HCM. Using supervised machine learning applied to training (n=137) and validation cohorts (n=61), this 7-biomarker panel differentiated HCM from healthy controls with an area under the curve of 1.0 in the training data set (sensitivity 100% [95% CI, 95–100]; specificity 100% [95% CI, 96–100]) and 0.82 in the validation data set (sensitivity 75% [95% CI, 59–86]; specificity 88% [95% CI, 75–94]). Reduced circulating levels of 4 other peptides (apolipoprotein L1, complement 5b, immunoglobulin heavy constant epsilon, and serum amyloid A4) found in children with high sudden cardiac death risk provided complete separation from the low and intermediate risk groups and predicted mortality and adverse arrhythmic outcomes (hazard ratio, 2.04 [95% CI, 1.0–4.2]; *P*=0.044).

**CONCLUSIONS:**

In children, a 7-biomarker proteomics panel can distinguish HCM from controls with high sensitivity and specificity, and another 4-biomarker panel identifies those at high risk of adverse arrhythmic outcomes, including sudden cardiac death.

Hypertrophic cardiomyopathy (HCM) is an inherited heart muscle condition characterized by left ventricular hypertrophy that can present throughout life. In adults, it is most commonly caused by dominantly inherited variants in genes encoding proteins of the cardiac sarcomere or *Z* disk.^1,2^ In infancy, the prevalence of malformation syndromes and inborn errors of metabolism is higher than in adults, but the etiology and clinical phenotype of older children with HCM are broadly similar to adult-onset HCM.^3^ Despite this, childhood-onset HCM is associated with greater lifelong morbidity and mortality related to sudden cardiac death (SCD) and heart failure.^4^ The advent of new treatments for patients with nonsyndromic HCM, including cardiac myosin inhibitors^5,6^ and nucleic acid therapies, represents an opportunity to modify or arrest disease development at an early stage of disease, but the efficient and safe use of these interventions in randomized clinical trials requires reliable predictors of disease development and prognosis.

In this regard, we previously used quantitative proteomics and supervised machine learning (ML) to develop a targeted multiple reaction monitoring liquid chromatography mass spectrometry-based assay in adult patients with HCM. This panel has diagnostic potential and is associated with the clinically estimated HCM-SCD risk score.^7^ The aim of this study was to explore the utility of this assay in childhood-onset HCM and to conduct an additional discovery on the proteomics plasma screen of children with HCM. The results were combined into a tier-2^8^ targeted proteomic assay, which was then tested in a separate prospective validation cohort of childhood patients with HCM and controls to test its association with adverse cardiac events.

## METHODS

With publication, all de-identified MS proteomics data will be openly available via the ProteomeXchange Consortium (http://proteomecentral.proteomexchange.org) and the PRIDE partner repository^9^ (PRIDE Accession Number: PXD045304). Source codes are available via GitHub (https://github.com/gcaptur/Paediatric-HCM-Proteomics).

All recruited participants provided written informed consent conforming to the Declaration of Helsinki (fifth revision, 2000). The UK National Research Ethics Service approved the generic analysis of de-identified clinical scans.^10^ The local research ethics committee gave approval for both proteomic method development on plasma specimens and plasma collection and sampling for this study (research ethics committee number: 15/LO/1888; UK Integrated Research Application System number: 185164).

The full methods are available as Supplemental Data.

### Statistics

Statistical analysis was performed in R^11^ (version 4.1.2). The distribution of data was assessed on histograms and using the Shapiro-Wilk test and Q-Q plots. Continuous variables are expressed as mean±1SD, or median or interquartile range; categorical variables are expressed as counts and percentages. Difference tests for continuous data were the Student’s *t* test or Mann-Whitney *U* test for parametric and nonparametric unpaired groups, respectively, and implemented Holm’s correction where appropriate. A sensitivity analysis was performed, repeating the difference tests for diagnostic biomarkers in solely sarcomeric HCM versus controls after removing patients with syndromic HCM and those with inborn errors of metabolism. The contingency table difference tests were performed by Fisher’s exact test. Correlations were calculated using Spearman’s ρ. Optimal cutoff values to discriminate between patient groups for each plasma biomarker separately were obtained using R package cutpointr maximize metric function, and area under the curve was also individually estimated. The best biomarker panel for HCM was built using supervised ML with the support vector machine (SVM) classification as previously described.^12^ Briefly, SVMs are a set of effective, supervised nonparametric ML techniques that analyze data and recognize patterns and are especially suited to 2-group separation challenges^13,14^ like the present one. The goal of our SVM model was to use the proteomics biomarker panel to predict which phenotypic category a participant belonged to (HCM or control) based on an initial training set example. We therefore used SVM to build a classifier by creating a decision boundary between the HCM and control classes that enabled the prediction of labels from 1 or more feature vectors, in this case the plasma proteomics biomarkers. This decision boundary, also known as the hyperplane, is oriented such that it is as far as possible from the closest data points from each of the classes.^15^ These closest points are the support vectors. We used the kernel SVM algorithm to be able to model higher-dimensional, nonlinear data. To overcome the problem of imbalanced classification due to the smaller number of controls versus cases in our cohorts,^16^ random oversampling of the minority class was used, thus avoiding information loss (R package ROSE^17^). The resultant balanced case-control data set was then randomly split into training and validation data sets (ratio 3:1). The performance of the tuned SVM was verified in the validation data set, and overfitting was avoided through the implementation of a 10-fold cross-validation (R package E1071). We constructed the SVM with a radial kernel tuned to cost 10 and gamma 2 to derive ML prediction scores per participant. For the optimal model, the area under the receiver operating characteristic curve was calculated using package ROCR. Sensitivity analysis against overfitting was undertaken by both undersampling and oversampling the imbalanced data and then repeating the SVM. Receiver operating characteristic curve was also used to determine the optimal cutoff values of prognostic biomarkers for predicting adverse outcomes during follow-up. Survival in patients with high versus low biomarker abundance was evaluated using the Kaplan-Meier method and compared among groups using the log-rank test. The index date was the date of recruitment, that is, the date of sample collection. Hazard ratios (HRs) are expressed as mean±95% CIs. *P* values are 2-sided and considered significant when <0.05.

### Outcomes Analysis

The arrhythmic end point during follow-up was any of cardiovascular death, ventricular fibrillation cardiac arrest, sustained ventricular tachycardia, or primary prevention implantable cardioverter defibrillators (ICD) fitted during the follow-up period (indicating a step change in the SCD risk estimate during follow-up).

### Clustering Analysis

Levels of peptides in the form of nonparametric continuous data were *Z* score normalized before principal component analysis (PCA). PCA was done using R package factoextra to extract and visualize PCA elements. Hierarchal clustering analysis of *Z* score normalized data was performed using function hclust, R/bioconductor package ComplexHeatmap, and k-means clustering.

### Pathway Analysis

Proteins from the MS data set were inputted into PathfindR along with their respective log_2_ fold change and false discovery rate value,^18^ and the KEGG pathway database was used (https://www.genome.jp/kegg/pathway.html). Only pathways that had a *P* value of ≤0.05 were considered. We implemented hierarchical clustering using a pairwise distance matrix based on the kappa statistics between the enriched terms. The active subnetwork search requires a minimum of 10 imputed genes. The final table produced by PathfindR includes a table of significant pathways with an associated adjusted *P* value, a fold enrichment value of the pathway, the lowest and highest *P* values generated from each iteration of the pathway analysis, and the proteins with increased or decreased abundance from the input protein list for every pathway.^18^

### Protein Interaction Network Analysis

A human protein interaction network was constructed for the promising diagnostic and prognostic HCM biomarkers using interaction data gathered from the IntAct App^19,20^ (release 1.0.0) and filtered to remove nonhuman nodes and interactions, interactions with chemicals, self-loops, and duplicated edges. Cytoscape^21^ (version 3.10.0) was used as a visualization tool. The data presented in Figure S1C are from an advanced IntAct Exact Query limited to the species *Homo Sapiens*. The final interactome topology was cross-checked with that generated in Interactome3D.^22^ The complete list of proteins and interaction metadata is provided in Data File S2.

### Role of Funders

The funders had no role in study design, data collection, data analysis, data interpretation, or writing of the report.

## RESULTS

### Study Participants

**Figure 1.**
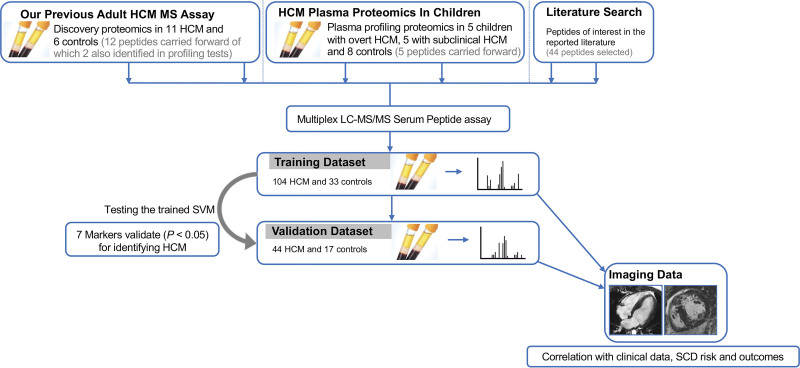
**Study consort diagram.** HCM indicates hypertrophic cardiomyopathy; LC-MS, liquid chromatography mass spectrometry; SCD, sudden cardiac death; and SVM, support vector machine.

Clinical and demographic characteristics of the cohorts are provided in Table 1. One hundred and ninety-eight children between the ages of 1 and 18 years were recruited (Figure 1), consisting of 148 children with HCM and 50 healthy controls (14 siblings of HCM probands free of the familial variant and 36 from the channelopathy clinic with normal imaging and electrocardiography and no channelopathy variants). Genetic variants identified in patients with HCM are summarized in Table 1, with full details provided in Table S1. Sixty children (41%) with HCM carried pathogenic/likely pathogenic sarcomere gene variants, while 37 (25%) had a diagnosis of syndromic HCM or HCM associated with inborn errors of metabolism.

**Table 1.**
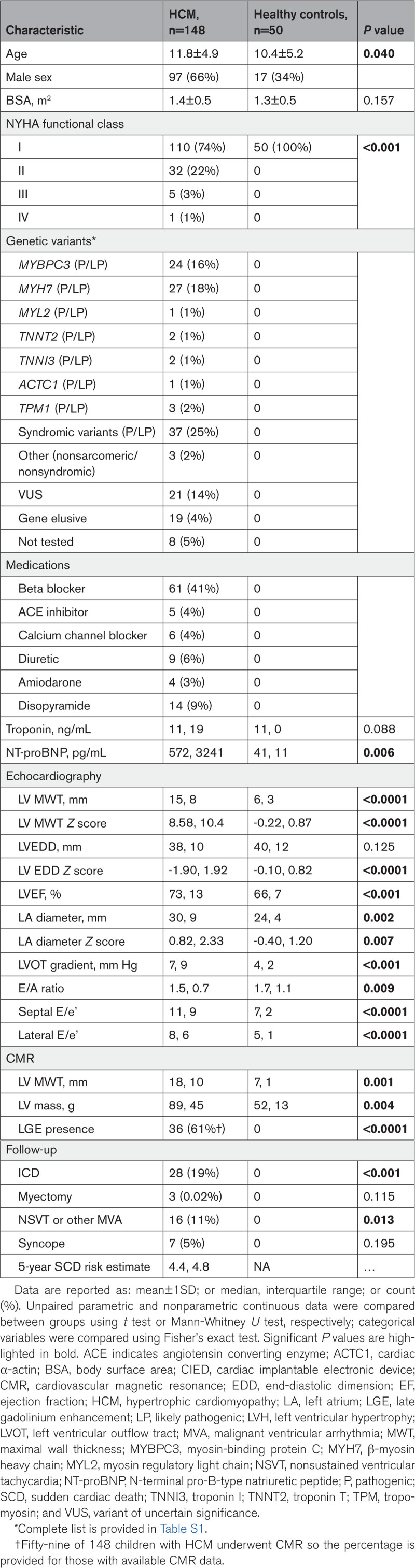
Baseline Characteristics of Study Participants

Controls were younger than patients with HCM (*P*=0.040), but there was no significant correlation between age and plasma levels of key proteomics markers in controls (all *r*_s_ *P*≥0.05). The training data set comprised n=104 HCM patients and n=33 controls (controls oversampled to n=96), while the validation data set comprised n=44 HCM patients and n=17 controls (controls oversampled to n=56).

### Association With Baseline HCM Phenotype

**Figure 2.**
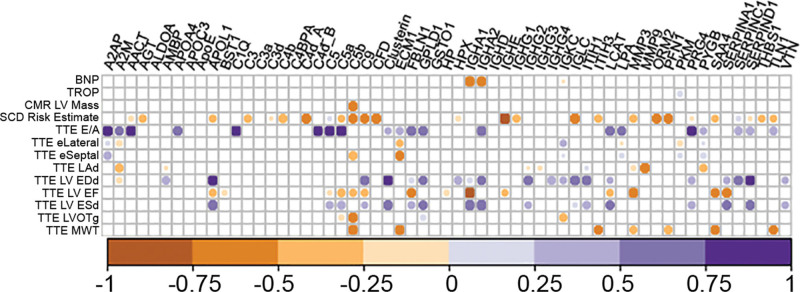
**Correlations of plasma proteomics marker levels with clinical/imaging traits in children with hypertrophic cardiomyopathy (HCM).** Correlogram of plasma levels of each of the 59 peptides (columns) with key clinical and imaging biomarkers (rows) in children with HCM. Only significant correlations (*P*<0.05) are shown. Circle size and colors reflect strength of the Spearman’s correlation coefficient; interesting positive and negative correlations are highlighted in purple/orange respectively. BNP indicates brain natriuretic peptide; CMR, cardiovascular magnetic resonance; E/A, E/A ratio; EDd, end-diastolic diameter; EF, ejection fraction; eLATERAL, lateral E/e’ ratio; ESd, end-systolic diameter; eSEPTAL, septal E/e’ ratio; LAd, left atrial diameter; LV, left ventricular; LVOTg, left ventricular outflow tract gradient; MWT, maximal wall thickness; TROP, troponin; and TTE, transthoracic echocardiography.

Continuous plasma proteomics biomarkers were non-normally distributed. Correlation analyses (Figure 2) showed that in children with HCM, plasma biomarkers TLN1 (talin-1), PYGB (glycogen phosphorylase B), and LPA (lipoprotein a) were positively correlated with echocardiographic markers of diastolic dysfunction (E/A ratio and septal E’, all *P*<0.05). In addition, plasma abundance of C5b (complement 5b-peptide) and SAA4 (serum amyloid A4-peptide) was negatively correlated with maximal wall thickness by echocardiography (*r*_s_, −0.71; *P*=0.013 and *r*_s_, −0.61; *P*=0.023, respectively); C5b was inversely correlated with LV mass by cardiovascular magnetic resonance (*r*_s_, −0.61; *P*=0.043), LV outflow tract gradient (*r*_s_, −0.64; *P*=0.004), and LV ejection fraction by echocardiography (*r*_s_, −0.36; *P*=0.018), as were APOL1 (apolipoprotein L1-peptide) and SAA4 (*r*_s_, −0.36; *P*=0.033, and *r*_s_, −0.54; *P*=0.013, respectively, with LV ejection fraction).

### 7-Biomarker Panel Distinguishes HCM From Controls

The results of the plasma proteomics profiling experiments used to develop the multimarker assay are reported in Table S2, and the provenance of the included biomarkers is explained in Data File S1.

**Figure 3.**
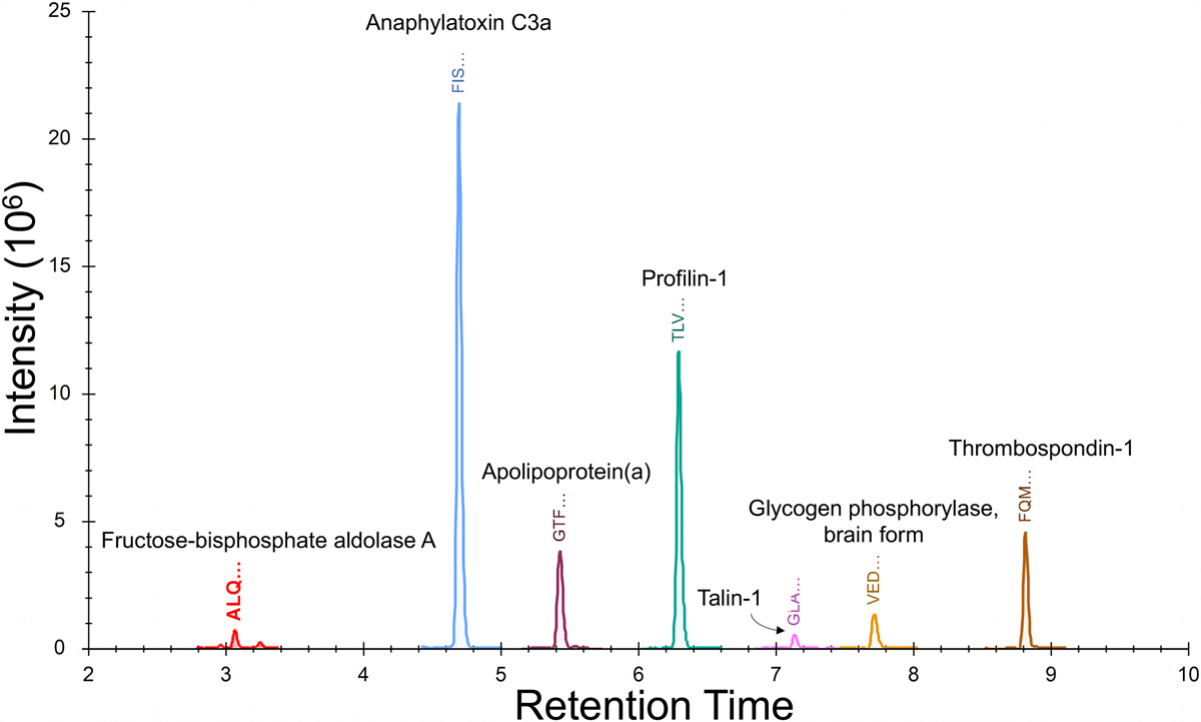
**Chromatogram showing the 7 diagnostic peptide biomarkers identifying hypertrophic cardiomyopathy (HCM) in children.** Overlaid chromatogram of the 7 marker peptides in the multiplexed targeted proteomic assay that showed diagnostic potential. C3a indicates complement 3a anaphylatoxin.

**Figure 4.**
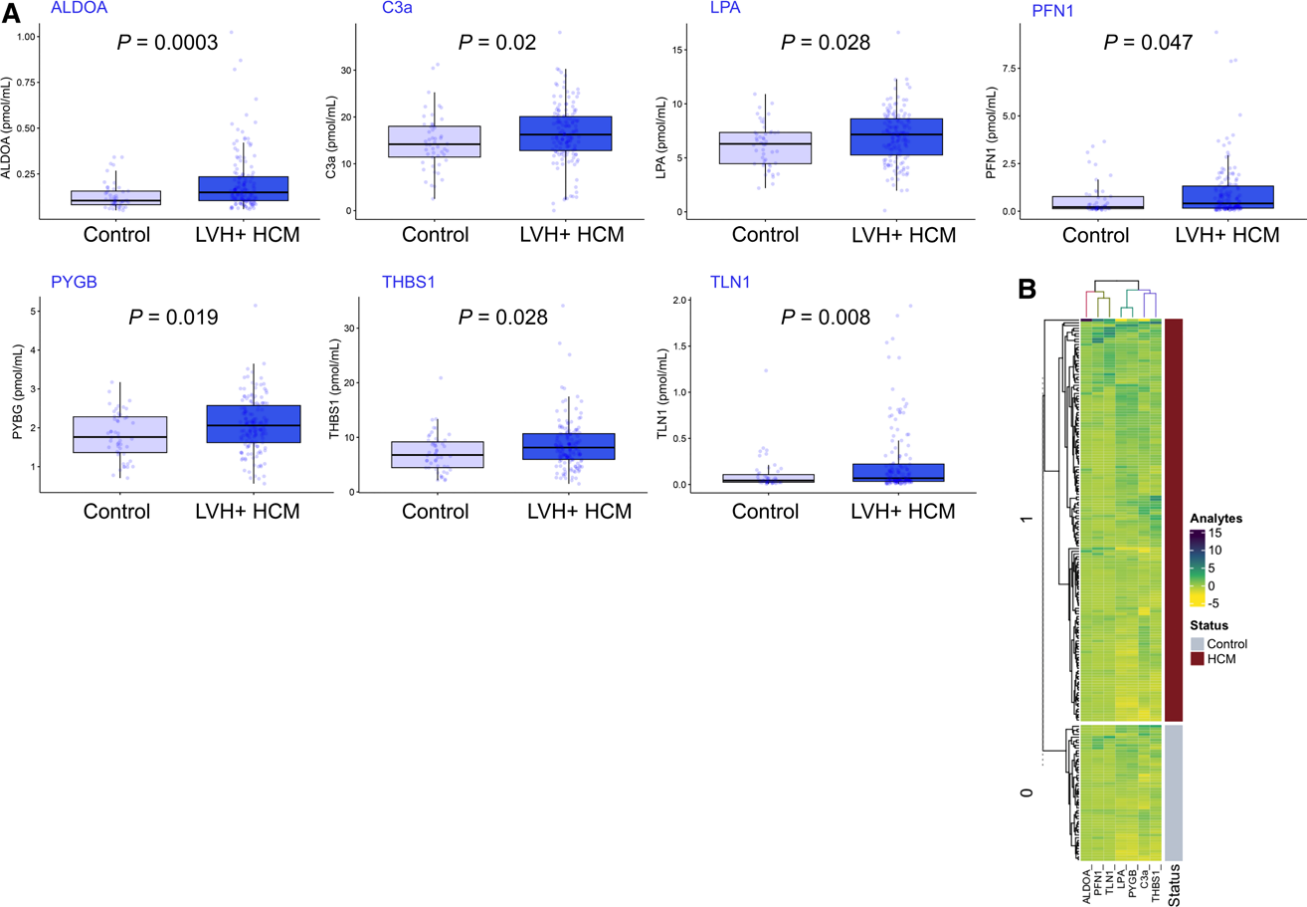
**Differentially abundant proteins between hypertrophic cardiomyopathy (HCM) and controls. A**, Box and whisker plots showing the 7 differentially expressed plasma peptides identified in the training data set consisting of HCM and controls, by the targeted proteomic multiplexed assay (using the Mann-Whitney-Wilcoxon test for nonparametric data with *P* value adjustment for multiple comparisons by the Holm method). **B**, Heatmap of these 7 differentially abundant proteins comparing all children with HCM (n=148) and controls (n=50), grouped and then clustered according to HCM status. Color bar represents range of normalized plasma concentration for each protein. Cluster 1 representing HCM expresses the highest levels of these proteomics biomarkers compared with controls. ALDOA indicates aldolase fructose-bisphosphate A; C3a, complement 3a anaphylatoxin; LPA, lipoprotein (a); PFN1, profilin 1; PYGB, glycogen phosphorylase B; THBS1, thrombospondin 1; and TLN1, talin-1.

**Figure 5.**
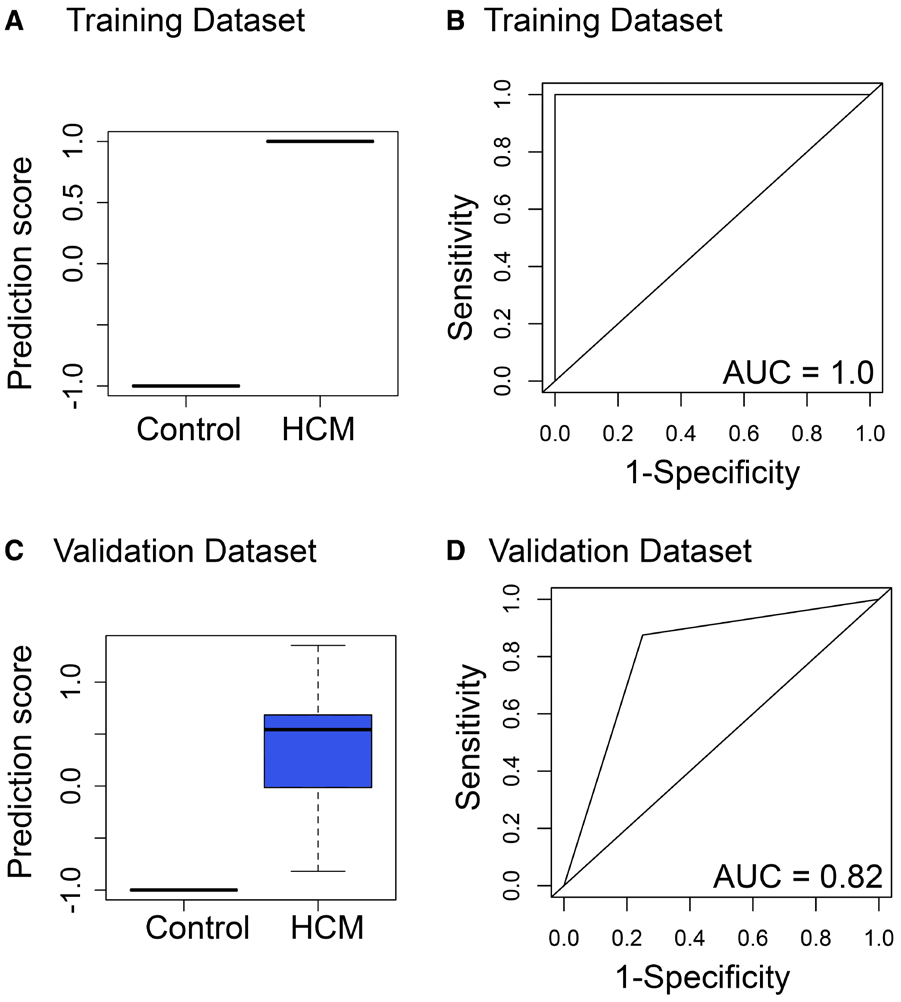
**Proteomic signature distinguishes hypertrophic cardiomyopathy (HCM) from controls in training and validation data sets.** Box plots showing performance of prediction scores calculated by a support vector machine supervised machine learning method in the training (**A**) and validation (**C**) data sets made up of children with HCM (n=104 and n=44, respectively) and controls (n=33 oversampled to 96 and n=17 oversampled to 56, respectively), including random oversampling to compensate for unbalanced classifiers (whiskers indicate variability outside the third and first quartiles [75th and 25th percentiles] represented as hinges around the median [bold midline]). Receiver operating characteristics (ROC) curves (**B** and **D**) show performance of the machine learning prediction score in training and validation data sets. AUC indicates area under the curve.

Targeted proteomic multiple reaction monitoring analysis of 59 candidate proteins (Data File S1) was performed blindly for 258 individual plasma samples over 3 days. Using this tandem mass spectrometry liquid chromatography mass spectrometry-based assay, 7 marker peptides (Figure 3; Table 2; Table S3) were found to be differentially abundant in the plasma of HCM cases compared with controls in the training data set (Figure 4). These 7 markers—ALDOA (aldolase fructose-bisphosphate A), C3a (C3 anaphylatoxin), LPA, PFN1 (profilin 1), PYGB, TLN1, and THBS1 (thrombospondin 1) —identified a HCM phenotype compared with controls, with an area under the curve of 1.0 in the training data set (sensitivity, 100% [95% CI, 95–100]; specificity, 100% [95% CI, 96–100]; Figure 5A and 5B; Table S4) and 0.82 in the validation data set (sensitivity, 75% [95% CI, 59–86]; specificity, 88% [95% CI, 75–94]; Figure 5C and 5D; Table S5).

**Table 2.**
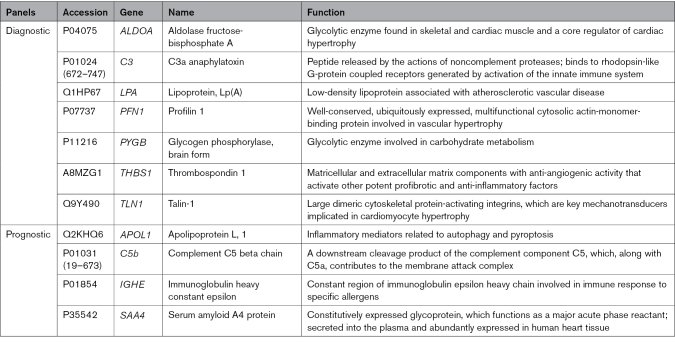
Summary of Diagnostic and Prognostic Biomarker Panels in Childhood-Onset HCM

In the sensitivity analysis using both undersampled and oversampled data, the 7-biomarker panel identified a HCM phenotype compared with controls, with an area under the curve of 1.0 in the training data set (sensitivity, 100% [95% CI, 98–100]; specificity, 100% [95% CI, 98–100]) and 0.98 in the validation data set (sensitivity, 99% [95% CI, 97–99]; specificity, 96% [95% CI, 93–98]).

In the sensitivity analysis considering sole sarcomeric HCM versus controls (after removing syndromic HCM and patients with inborn errors of metabolism), the majority of diagnostic biomarkers continued to show significant differences between groups (ALDOA, *P*=0.003; LPA, *P*=0.028; PYGB, *P*=0.033; TLN1, *P*=0.029), and where differences were attenuated (C3a, *P*=0.073; PFN1, *P*=0.133; THBSO, *P*=0.050), the trend for increased abundance in the children with sarcomeric HCM persisted.

### 4-Biomarker Panel Predicts SCD Risk and Outcomes

**Figure 6.**
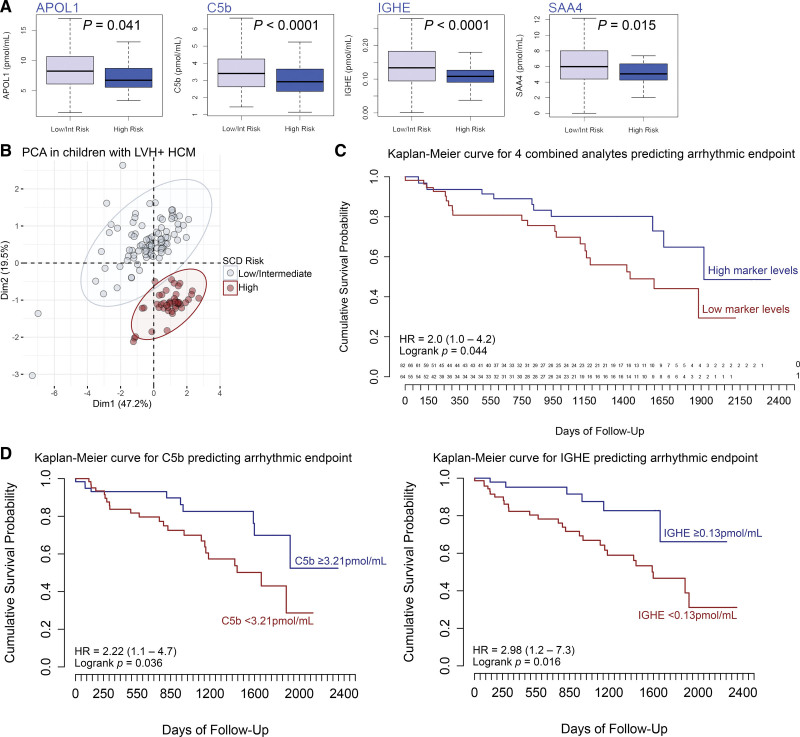
**Plasma proteomic signature identifies children with hypertrophic cardiomyopathy (HCM) at high risk of sudden cardiac death (SCD). A**, Four plasma markers were found to be reduced in children with HCM and high vs low/intermediate SCD risk (n=148): APOL1, C5b, IGHE, and SAA4 peptides. **B**, Principal component analysis plot of the plasma proteome in all children with HCM showing individual points colored by group reflecting their 5-year sudden cardiac death risk category, here stratified as high risk (≥6%; red) vs low/intermediate risk (<6%; gray). Concentration ellipses of children with high vs low/intermediate SCD risk diverge with complete separation of groups based on the abundance in plasma of these 4 circulating proteomics biomarkers. **C**, Survival curve showing the 4 combined proteomics markers predicting the arrhythmic end point and (**D**) individual survival curves for C5b- and IGE-peptides also predicting the primary arrhythmic end point. Remaining survival curves are provided in Figure S2. APOL1 indicates apolipoprotein L1; C5b, complement 5b; Dim, dimension; HR, hazard ratio; IGHE, immunoglobulin heavy constant epsilon; LVH, left ventricular hypertrophy; PCA, principal component analysis; and SAA4, serum amyloid A4.

Among the 148 children with HCM, 92 were classified as having a low or intermediate 5-year SCD risk (<6%), 45 had a high SCD risk (≥6%), and 11 were unclassified due to ≥1 missing parameters required for their algorithmic classification.^23^ Plasma abundance of 4 peptides (Table 2; Table S3) was significantly reduced in high versus low/intermediate SCD risk children as follows: APOL1, 7.49±2.94 versus 8.91±5.12 pmol/mL, *P*=0.041; C5b, 2.98±0.87 versus 3.68±1.51 pmol/mL, *P*<0.0001; IGHE (immunoglobulin heavy constant epsilon-peptide), 0.11±0.04 versus 0.15±0.09 pmol/mL, *P*<0.0001; and SAA4, 5.47±2.14 versus 6.68±3.56 pmol/mL, *P*=0.015 (Figure 6A). PCA of these plasma analytes revealed complete separation of cases (Figure 6B).

Of the 148 children with HCM, 1 was lost to follow-up, with the remainder being longitudinally followed-up for a median of 770 days (interquartile range, 182–1481).

During this period, 33 children reached the arrhythmic end point with 3 having died, 2 experiencing sustained ventricular tachycardia, and 28 receiving a primary prevention ICD during the follow-up period. In addition, 3 children underwent septal myectomy and 3 underwent heart transplantation.

Reduced abundance of C5b-peptide (HR, 2.22 [95% CI, 1.1–4.7]; *P*=0.036) and IGHE-peptide (HR, 2.98 [95% CI, 1.2–7.3]; *P*=0.016) predicted the arrhythmic end point (Figure 6A and 6B). The remaining survival curves for APOL1 and SAA4 are provided in Figure S2. The 4 analytes combined (APOL1, C5b, IGHE, and SAA4) also predicted the arrhythmic end point with a HR of 2.04 (95% CI, 1.0–4.2; *P*=0.044; Figure 6C). All prognostic biomarkers were negatively correlated with the 5-year SCD risk estimate (*r*_s_ between −0.31 and −0.93, *P*<0.039 all; Figure 2).

## DISCUSSION

**Figure 7.**
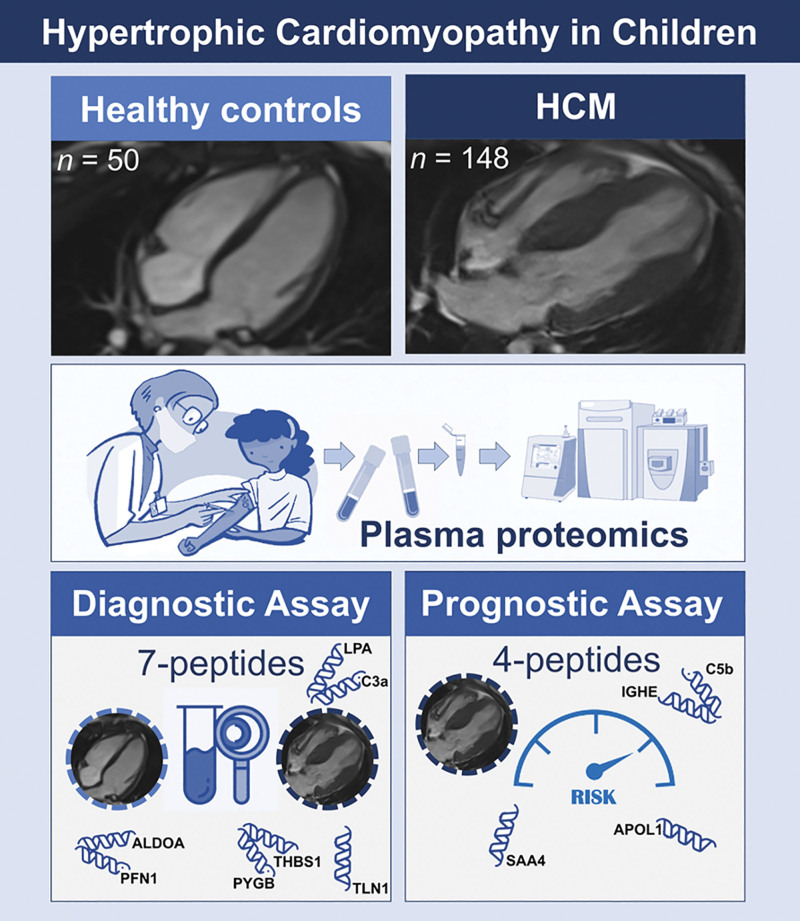
**Plasma proteomics biomarkers have diagnostic and prognostic potential in childhood-onset hypertrophic cardiomyopathy (HCM).** ALDOA indicates aldolase fructose-bisphosphate A; APOL1, apolipoprotein L1; C3a, complement 3a anaphylatoxin; C5b, complement 5b; HCM, hypertrophic cardiomyopathy; IGHE, immunoglobulin heavy constant epsilon; LPA, lipoprotein (a); PFN1, profilin 1; PYGB, glycogen phosphorylase B; SAA4, serum amyloid A4; and TLN1, talin-1,

In the present pediatric HCM study (Figure 7), we confirm the utility of a previously reported adult plasma proteomics diagnostic panel for HCM while identifying additional novel biomarkers. In children, the diagnostic 7-biomarker proteomics panel consisting of ALDOA, C3, LPA, PFN1, PYGB, TLN1, and THBS1 can identify HCM from controls with high sensitivity and specificity, while the 4-biomarker prognostic panel consisting of APOL1, C5b, IGHE, and SAA4 identifies children at high risk of SCD, predicting mortality and adverse arrhythmic outcomes.

### Biomarker Panels for Precision Disease Stratification

The concept of using protein biomarker panels for precision diagnostics and risk stratification in preference to individual protein biomarkers offers a more comprehensive representation of human physiology.^24^ Unlike traditional methods, this approach enables high-throughput, simultaneous analysis of panels of specific proteins with high specificity and sensitivity using minimal sample amounts. Although several proteomics methods potentially exist for quantifying such multimarker panels, many of them struggle to make inroads in clinical practice with only a handful of notable exceptions.^25^ On one hand, our targeted MS panels are more amenable to high-throughput clinical roll-out because of their low overall cost, high flexibility, independence from affinity reagents, and low entry barriers for their integration into existing laboratory workflows,^24^ given the almost ubiquitous availability of triple quadrupole MS platforms (mass spectrometers) in most tertiary hospital clinical laboratories. On the other hand, it can be challenging to implement proteomics panels in clinical routines and realize their full medical potential because of the issues of reproducibility, standardization and the need for extensive validation (and even revalidation) in broadly different cohorts globally that we recognize as our key priorities for future work. Another challenge with novel multimarker plasma proteomics tests is the need to define discrete pmol/mL concentrations for each marker that delineates diagnosis or prognosis, in addition to demonstrating the clinical utility of the combined panel. In the current proteomics study, as expected, the ML multimarker panel outperformed individual analytes for both diagnosis and prognosis (Table S3). The final acid test would be to retrospectively subject such proteomics panels to meta-analysis using individual patient-level data.^26^

### Putative Pathophysiological Mechanisms of Pediatric Plasma Proteomic Biomarkers

A detailed discussion of key diagnostic and prognostic HCM biomarkers identified in the present study is provided in the Supplemental Material and summarized in Table 2. Discovered diagnostic and prognostic plasma biomarkers may be epiphenomena indicative of a disease state without necessarily being causative. Broadly, the 7 diagnostic HCM biomarkers uncovered in the current study clustered around: fluid sheer stress and platelet activation pathways pertinent to the problem of LV outflow tract obstruction in HCM; atherosclerosis, which has a well-established association with inflammation; and the Rap1 (a rat sarcoma [RAS]-like small GTPase) and PPAR (peroxisome proliferator-activated receptors) signaling pathways, both of which have been implicated in the pathophysiology of inappropriate cardiomyocyte hypertrophy.^27,28^ This 7-biomarker panel included the same 4 analytes (ALDOA, C3, TLN1, and THBS1) that we had previously found to be diagnostic in a separate adult HCM cohort.^7^ That 6-biomarker adult HCM diagnostic assay^29^ also included RSU1 peptide (Ras suppressor protein 1-peptide)—a molecular scaffold for cellular focal adhesion—but RSU1 was undetectable in the current pediatric plasma profiling experiments and therefore not included in the 59 multimarker pediatric assay. It is plausible that RSU1 (which tracked plasma levels of NT-proBNP (N-terminal pro-B-type natriuretic peptide) in adult HCM^29^ and was elevated in the myocardium of mice with severe LV outflow tract obstruction^30^) is a latent plasma feature of HCM only occurring with more advanced disease, explaining its low levels in the plasma of children. Another adult HCM diagnostic biomarker—GSTO1 peptide (glutathione S-transferase omega-1)—though differentially abundant in children with HCM versus controls (*P*=0.002), was insufficiently discriminant compared with other markers to justify its inclusion in the final diagnostic panel by the SVM.

In children with HCM, we found a complex inflammatory state characterized by a diagnostic abundance of C3a compared with controls (Figure 4) and a relative depletion of C5 in affected children with high SCD risk and adverse outcomes (Figure 6). Several hypotheses have been proposed to explain the link between HCM and aberrant systemic inflammation, with myocardial fibrosis, cardiomyocyte injury, and subendocardial ischemia all implicated.^31^

### Clinical Implications of Novel Pediatric 7-Biomarker Diagnostic Panel

Our 7-biomarker diagnostic panel for childhood-onset HCM could potentially have a role as a surrogate end point in clinical drug or interventional trials^32^ to guide response to therapy, although further large-scale tests in sarcomeric versus nonsarcomeric HCM cohorts would be needed first. Such precision diagnostics based on proteomics are needed over and above currently available imaging, to better support recent advancements in precision therapeutics for HCM, particularly with the advent of myosin ATPase inhibitors, cardiac mitotropes, and gene therapies.^33^ Targeted plasma proteomics assays like ours have the advantage of being noninvasive with small blood aliquot requirements. They therefore open the door to the possibility of dried blood spot collections^34^ in children from a finger or heel prick that can be done at the child’s home^35^ by family members with minimal training, then to be posted to the MS laboratory. In the future, such remotely delivered high-throughput diagnostic assays would especially benefit infants and children by potentially reducing travel to tertiary cardiac centers for serial imaging, thereby reducing psychological distress^36^ and school absenteeism during sensitive educational years.^37^ Regular home-based serial proteomics monitoring of children with subclinical HCM (ie, sarcomere gene mutation carriers before the development of left ventricular hypertrophy) could potentially capture, with better granularity, the transition to disease, while in overt HCM it could better capture disease progression, response to exercise, and drug-induced reversibility. Contingent on such patient-level serial proteomics analysis is the need to understand discrete concentration cutoffs per analyte to definitively conclude in the first instance that a transition to disease has occurred and, in the second instance, to appreciate disease progression over time.

### Novel Pediatric 4-Bioimarker Prognostic Panel

The novel 4-biomarker prognostic panel that predicted mortality and adverse arrhythmic outcomes in childhood-onset HCM was able to distinguish patients with high versus low/intermediate 5-year SCD risk without misclassifications. Such a plasma-based risk tool for childhood-onset HCM could help select which children to prioritize for expedited imaging or closer clinical surveillance. Given that SCD is the most common mode of death in pediatric HCM^38^ and in light of recent Sarcomeric Human Cardiomyopathy Registry data showing a greater risk of life-threatening ventricular arrhythmias in childhood-onset compared with adult-onset HCM,^4^ the development of novel alternative risk stratification tools for pediatric HCM is highly encouraged.

Primary prevention ICD implantation that was dictated by the HCM Risk-Kids estimated SCD risk^23^ comprised the bulk of our secondary outcomes, and we found a substantial rate of ICD implantations in our cohort during the 6-year maximum follow-up period (28/148; 19%). This is in keeping with reported rates of ICD implantation in large multicenter pediatric HCM cohorts.^38^

### Limitations

This was a single-center study, albeit a national referral center in the United Kingdom. A larger, multicenter study is needed for verification of findings, encouraged by the fact that a plasma proteomics panel developed for adult HCM in an external cohort from another tertiary center^7^ also contained several of these pediatric HCM biomarkers. The pediatric HCM profiling experiment was performed on a small discovery cohort that only included patients with MYBPC3 (cardiac myosin-binding protein C) mutations; however, we mitigated this by including several markers from the earlier adult panel and a large number of candidate peptides from the published literature. Other diseases with hypertrophy and other cardiomyopathies were not explored, nor was histology, so these are targets for future work. The training and validation approach needed to permit supervised ML, meant that the final validation cohort was not large. Due to the inherent challenge of recruiting pediatric controls into clinical research studies involving blood donation and imaging tests, the cohort was imbalanced with a limited number of controls (n=50). However, we mitigated against the problem of unbalanced classification using lossless oversampling and have shown in the sensitivity analysis that a combined over/undersampled approach still generated similar results. Our cohort of patients with childhood-onset HCM (ages 1–18 years) was of heterogeneous genetic etiology and included sarcomeric HCM, syndromic HCM, and gene-elusive HCM, so the results are not immediately generalizable to infant-onset or early adulthood-onset HCM subgroups, which seem to have distinct natural histories and clinical courses based on longitudinal cohort data.^4^ Controls recruited from the channelopathy clinic might still have carried other ion channel variants missed by the standard targeted panels used.

### Conclusions

Using quantitative proteomic approaches in children, we have developed 2 translational and clinically useful plasma biomarker panels for HCM. Prognostic biomarkers correlate with the estimated risk of SCD and predict major arrhythmic cardiovascular events. Findings may provide novel ways to refine disease monitoring and improve risk stratification in pediatric HCM.

## ARTICLE INFORMATION

### Acknowledgments

The authors gratefully acknowledge all the participants consenting to this research and for donating their samples and data for these analyses, and the research teams involved in consenting, recruitment and sampling of participants.

### Sources of Funding

This study was supported by an Action Medical Research/LifeArc Project Grant to Profs Kaski and Mills. Dr Barnes, E. Field, and Prof Kaski are supported by Max’s Foundation and Great Ormond Street Hospital Children’s Charity. Prof Kaski is supported by a Medical Research Council (MRC) Clinical Academic Research Partnership (CARP) award (MR/T024062/1). Dr Captur is supported by the British Heart Foundation (BHF, SP/20/2/34841), the BHF Accelerator Award (AA/18/6/34223), the National Institute of Health Research (NIHR) Invention for Innovation (i4i) Funding at the Speed of Translation (FAST) grant scheme (NIHR205960), Barts Charity HeartOME Grant, and the NIHR University College London (UCL) Hospitals Biomedical Research Centre (BRC). Prof Mills and Dr Heywood are supported by the NIHR BRC at Great Ormond Street Hospital for Children National Health Service Foundation Trust and UCL.

### Disclosures

The authors declare an existing UK patent on the adult hypertrophic cardiomyopathy proteomics panel GB1815111, for a novel high-throughput, multiplex, targeted proteomic plasma assay.

### Supplemental Material

Supplemental Methods

Figure S1 and S2

Tables S1–S5

Data File S1 and S2

References 39–77

## Supplementary Material


