# Supplementary material for: Novel Multiplexed Plasma Biomarker Panel Has Diagnostic and Prognostic Potential in Children With Hypertrophic Cardiomyopathy
Source: Circ Genom Precis Med. 2024 Jun 7;17(3):e004448. doi: 10.1161/CIRCGEN.123.004448 (PMC11188636; doi:10.1161/CIRCGEN.123.004448)

SUPPLEMENTAL MATERIALS

Table of Contents

Supplemental Methods 2
Supplemental Tables 5
Table S1. Genetic status of children with HCM recruited into the study. 5
Table S2. Differentially abundant peptides between HCM and controls in the HCM plasma profiling experiments. 6
Table S3. Optimal cut-offs for biomarkers considering single analytes as opposed to multi-marker panels. 7
Table S4. Confusion matrix for the SVM analysis of training dataset. 8
Table S5. Confusion matrix for the SVM analysis of validation dataset. 8
Supplemental Figures 9
Figure S1. Ranked pathway analysis for abundant proteins in HCM and human interactome. 9
Figure S2. Additional survival curves for arrhythmic endpoint that did not reach statistical significance. 10

Supplemental Methods

Study design and population. This was an observational, prospective, longitudinal study of children who were recruited from a dedicated cardiomyopathy clinic at the Centre for Paediatric Inherited and Rare Cardiovascular Disease, at Great Ormond Street Hospital, London, UK national referral centre.

Two cohorts were recruited: 1) children with genetically heterogenous HCM and 2) healthy controls consisting of siblings of children with HCM free of the familial disease-causing variant, and children with structurally normal hearts, normal electrocardiograms and free of disease-causing variants clinically referred for imaging from the channelopathy clinic, as part of family screening.

Diagnosis of HCM was based on the demonstration by echocardiography or cardiovascular magnetic resonance (CMR) of a hypertrophied LV with a maximal wall thickness (MWT) of ≥2 standard deviations (SD) above the body surface area-corrected paediatric population mean (*z* score ≥2) that could not be explained solely by abnormal loading conditions or in accordance with published criteria for familial disease[1]. Children with known inborn errors of metabolism or syndromic causes of HCM were also included in the HCM cohort. Exclusion criteria for all participants were needle-phobia prohibiting blood sample collection and a recent history (<1 month) of blood transfusion or haemodialysis. Estimates for five-year risk of SCD were calculated using the validated paediatric HCM Risk-Kids tool[2]. Patients were categorized as having low (<4%), intermediate (≥4<6%) or high (≥6%) five-year risk of SCD[3].

Sample collection. Whole blood was collected from each child in one 4.9 mL S-Monovette® EDTA K3E tube, containing ethylenediaminetetraacetate tripotassium. Tubes were inverted 5 times at the time of blood collection and centrifuged on-site at 22 °C for 15 minutes at 2200xg to separate supernatant from buffy coat and blood cells. The serum was collected using a transfer pipette and aliquoted into 1–5, 500ul-2ml cryovials (according to the volume of each sample). Aliquoted plasma samples were stored in a freezer at −80 °C until use, at which point a single aliquot was thawed to permit the withdrawal of ten microlitres (10 µl) of plasma for profiling analysis or 30 µl for targeted MRM-LC-MS/MS analysis. Briefly, peptides were trapped and desalted prior to reverse phase separation using a precolumn. Peptides were then eluted off the precolumn and separated on the analytical column. Label free proteomic profiling samples were depleted using Pierce Top 14 Abundant Protein depletion spin (Thermo Fisher Scientific) columns, while the targeted analysis depletion was done using ammonium sulfate precipitation as previously described[4]. Additionally, participants donated blood samples for measurement of N-terminal prohormone of brain natriuretic peptide (NT-proBNP) and troponin levels, and for genetic analysis.

Genetic analysis

Genetic sequencing methods varied according to year, type of test requested (diagnostic or predictive) and individual laboratory conducting testing. Targeted testing of HCM genes was performed using direct Sanger sequencing (3-11 genes) prior to 2011. After 2011, next-generation sequencing was more widely available (21-104 gene panels). Pathogenicity of variants was reclassified using current American College of Medical Genetics (ACMG) guidelines. Variants affecting the sarcomere genes were classified as either thick-filament (myosin-binding protein C [*MYBPC3*], β-myosin heavy chain [*MYH7*], myosin regulatory light chain [*MYL2*]) or thin-filament variants (troponin T [*TNNT2*], troponin I [*TNNI3*], tropomyosin [*TPM1*], cardiac α-actin [*ACTC1*]).

Proteomic analysis

HCM plasma label free proteomics analysis. To identify candidate plasma biomarkers for HCM an additional smaller group of children was recruited at study start, separate from the main HCM and control cohorts previously described. Ten µL of plasma were thus collected from 8 healthy controls and 5 children with HCM carrying a *MYBPC3* gene variant (mean age in controls 11.0±5 years vs 14.4±4 years in patients, p=0.118). Plasma samples were depleted for high abundance proteins using Top 14 depletion columns (Thermo, UK). Eluted depleted protein concentration was determined using a protein bicinchoninic acid (BCA) assay[5]. Protein was fractionated by high pH low pH method as previously described[6,7]. Fractions were then analyzed using MS^E label-free quantitation as previously described[8]. In plasma profiling experiments comparing healthy control samples with those from children with HCM, 5 proteins were found to be differentially abundant (all $p<0.05$, **Table S2**) and therefore included in the multiplex panel. The complete list of peptides interrogated as part of the profiling experiments is provided in the sheet '**Profiling**' in **Data_File_S1**

Peptide selection for tandem mass spectrometry assay. Potential biomarkers identified through plasma profiling were combined with those previously identified in a separate HCM myocardial tissue proteomic profiling experiment undertaken in adults[9] and from a literature search of published proteomics panels. These combined proteotypic peptides were then developed into a multiplexed and targeted proteomic plasma test (**Supplemental Data File S1**) that was applied to all study members in the current cohort. Assays for newly identified markers were set up using quantotypic peptides ideal for multiple-reaction-monitoring (MRM) method application. These were selected using open access databases the gpm.org and Peptide Atlas. The top three most observed tryptic peptides that were unique were selected for each biomarker candidate and assessed in pooled serum. Only peptides that gave stable (low coefficients of variability %) quantitative data, and good signal-to-noise ratio of the endogenous peptide, were used in the assay. Two transitions per peptide were selected for inclusion in the final multiplex assay: one for quantitation and one for confirmation. The most abundant clean transitions without interfering non-specific peaks were selected.

MRM-LC-MS/MS assay sample preparation. In-house method for albumin depletion was performed on 30 µl of plasma. Briefly, 10 ul of Heavy labelled QconCAT protein (PolyQuant, Germany, 0.5 pmol/ul) was added to the sample and vortexed. To that 60 µl of 3M Ammonium Sulphate was added reducing the concentration to 1.8 M. Samples were sonicated for 15 minutes (sonicator bath) and centrifuged for 30 minutes at 500 g. Supernatant was discarded and the pellet washed with addition of 200 ul of 1.8 M Ammonium Sulphate. Pellets were centrifuged again and supernatant removed. Protein pellets were re-suspended in 20 µL of 200 mM Tris, 1% amidosulfobetaine-14 (ASB-14), pH 7.8, containing 6 M urea, 2 M Thiourea and agitated at room temperature for 60

min. Disulfide bridges were reduced by the addition of 3 µL of 200 mM tris-hydrochloride (HCL), pH 7.8 containing 20 mM 1,4-dithioerythritol (DTE) and incubated at room temperature for 60 min. Free thiol groups were carboamidomethylated by incubation with 6 µL of 200 mM tris-HCL, pH 7.8 containing 20 mM iodoacetamide and incubated in the dark at room temperature for 45 min. The reaction mixture was then diluted with 155 µL of water and vortexed, and 1 µg of Trypsin Gold (Promega, UK) was added to the solution. Samples were incubated overnight (10 h) at 37 °C in a Thermomixer. Digested peptides were cleaned and desalted using C18 Bond Elute (Agilent UK) as described previously[10].

MRM LC-MS/MS analysis. Dried peptides were re-suspended in 50 µL 5% acetonitrile | 0.1% trifluoroacetic acid (TFA). Five microlites (5 µl) of each sample were injected into a Xevo TQ-XS triple quadrupole mass spectrometer coupled to a Waters I class Acquity ultra-performance liquid chromatography (UPLC) system (Waters PLC, Manchester UK). The instrument was operated in positive ion mode. The capillary voltage was maintained at 2.8 kV with the source temperature held constant at 150 °C. A Waters Acquity UPLC Premier Peptide column 1.7 µm 2.1 x 50 mm pre-column was used for separation with solution A (LC-MS grade water with 0.1% formic acid) and solution B (LC-MS grade acetonitrile with 0.1% formic acid). The flow rate was set to 0.3 ml/min and a linear gradient of 0 to 30% solution A over 7 min. Gradient was increased to 100% B for 1 minute and held for one more. Initial conditions were restored at 10 minutes and column was equilibrated for next injection The total run time was 12 min. Pooled plasma digest from all samples was used as a quality control (QC) which was run periodically between injections. QCs were monitored throughout the run and coefficients of variation below ±15% were recorded and considered acceptable. Chromatograms were analyzed using SkyLine software (MacCoss labs, USA). Semi-automated peak integration was processed by manual inspection to correct for false assignments. The reader was fully blinded to the clinical status of study members throughout the analysis procedure. Integrated peak areas were *z*-score normalised across analytes for subsequent analysis. The MS proteomics data have been deposited to the ProteomeXchange Consortium via the PRIDE [11] partner repository with the PRIDE Accession Number: PXD045304.

Cardiac imaging

Transthoracic echocardiography. Patients with HCM were evaluated by standard 2-dimensional and Doppler transthoracic echocardiography in accordance with previously published methods[12]. Recorded variables included: left atrial diameter on parasternal long axis view, LV end-systolic dimension, LV end-diastolic dimension, MWT, and LV outflow tract gradient at rest and with Valsalva. MWT was defined as the greatest thickness in any single LV segment measured at end-diastole in the parasternal short-axis plane at either the level of the mitral valve, mid-ventricle or apex. LV volumes, ejection fraction and diastolic parameters were measured according to established guidelines[13,14]. Briefly, LV volumes and ejection fraction were assessed by biplane Simpson's equation using the apical 4- and 2-chamber views. Pulsed Doppler sample volume (1–3 mm) was placed on the edge of the mitral leaflets to determine mitral diastolic flow properties in the apical 4-chamber view. The peak flow velocity of early diastolic E-wave and late diastolic A-wave, E/A ratio, and E velocity deceleration times were recorded. Tissue Doppler imaging in the apical 4-chamber view, with sample volume at the septal and lateral segment of the mitral annulus provided e′ values.

Cardiovascular magnetic resonance imaging. A subset of controls and patients with HCM free of cardiac implantable electronic devices, permanent atrial fibrillation and without breath-holding difficulties at baseline, underwent CMR scanning. Standard clinical scans were performed using a 1.5 Tesla scanner (Avanto, Siemens Medical Solutions, Erlangen, Germany). CMR short axis volumetric studies[15] were acquired as cine steady-state free precession including long-axis cines in the two-, four- and three-chamber views[16]. Standard late gadolinium enhancement (LGE) images were acquired using a fast low angle single shot inversion recovery sequence following a contrast bolus of 0.1 mmol/kg of gadoterate meglumine (Dotarem; Guerbet, Paris, France) and analyzed for presence/absence of LGE by one experienced observer with >3 years of experience in CMR, using OsiriX MD[17].

Description and function of diagnostic proteomics biomarkers for HCM

Aldolase Fructose-Bisphosphate A (ALDOA) is a glycolytic enzyme found in skeletal and cardiac muscle[18] and a core regulator of cardiac hypertrophy[19]. The current study found an increased abundance of ALDOA in the plasma of children with HCM compared to controls, which tallies with our previous study findings in adult-onset HCM. Animal studies have also shown that ALDOA activity is up-regulated in hypoxic tissues,[2] raising the possibility that subendocardial ischaemia in HCM is underpinning the high ALDOA levelsin our patients.

C3 anaphylatoxin (C3a) is a peptide released by the actions of non-complement proteases and it binds to rhodopsin-like G-protein coupled receptors generated by activation of the innate immune system. We found that a peptide containing C3 peptide with the anaphylatoxin sequence was increased in patients with HCM compared to controls. Our data cannot confirm it is specifically the proteolytically processed activated anaphylatoxin that was increased as the tryptic peptide could also be present in unprocessed C3 but other C3 peptides without the anaphylatoxin sequence were not affected therefore it is a possibility that the C3 peptide detected in our data derives from increased C3 anaphylatoxin in the plasma. Several other clinical studies have similarly found high levels of related proteins from the complement cascade in the plasma of patients with HCM or other forms of LVH, including our own previous adult HCM proteomics study[9]. In the present study, we also found increasing plasma levels of complement component C5 tracked phenotype evolution in HCM, with strong trajectory similarities for evolving diastolic dysfunction, obstruction and left atrial dilatation. Interestingly, in the prognostic analysis, we found that a peptide containing Complement C5 beta chain (C5b) with the anaphylatoxin sequence was reduced in high risk HCM patients when compared to their low-risk counterparts. This tallies with experimental murine data showing how C5-deficiency might predispose mice to cardiac dysfunction on account of depressed cardiac metabolism and maladaptive reactivation of the so-called "fetal gene program"[21]. Fetal gene reactivation is widely accepted to be a sign of cardiac stress and pathology, since physiological hypertrophy alone does not induce their expression[22]. It is therefore plausible that the declining levels of C5b in children with HCM, heralds fetal gene reactivation putting them at higher risk of suffering adverse cardiac events.

Lipoprotein(a) (LPA) is a type of low-density lipoprotein (LDL) known for its association with atherosclerotic vascular disease. Oxidized LDL has also been shown to increase mechanical stress-induced cardiac hypertrophy in mice[23], potentially mediated through the upregulated expression of Angiotensin II receptor type 1 (AT1) which is implicated in the hypertrophic responses.
Profilin 1 (PFN1) is a well-conserved, ubiquitously expressed, multifunctional cytosolic actin-monomer-binding protein[24], involved in vascular hypertrophy. Recent data also point to its role as a potential mediator of cardiomyocyte-specific hypertrophic remodelling and it has been shown to be elevated in multiple mouse models of hypertrophy[25]. Indeed in animal studies, overexpression of PFN1 is sufficient to induce cardiomyocyte hypertrophy and sarcomeric remodelling, while silencing of PFN1 attenuates the hypertrophic response[26].
Glycogen Phosphorylase B (PYGB) is a glycolytic enzyme that plays an essential role in the regulation of carbohydrate metabolism and it may also be a sensitive biomarker of myocardial ischemia resulting from tissue hypoxia[27]. PYGB exists associated with glycogen in a macromolecular complex that is structurally bound to the sarcoplasmic reticulum. With the onset of tissue hypoxia, glycogen is broken down completely, PYGB converts from a structurally-bound to a soluble form, and so becomes free to move around in the cytoplasm and to diffuse out of the cell. In acute myocardial ischemia, the release of PYGB from the cardiomyocyte into the blood stream is the result of the concomitant glycogenolysis burst and increased plasma membrane permeability[28]. A clinical study in adults with HCM showed that plasma levels of PYGB was significantly raised in patients with HCM suggesting a potential role as biomarker of subclinical myocyte injury[29].
Thrombospondin 1 (THBS1) is a matricellular, non-structural extracellular matrix component with anti-angiogenic activity that is able to activate transforming growth factor-β, a potent profibrotic and anti-inflammatory factor.[30] In our adult plasma proteomics study, THBS1 was found to be diagnostic of HCM and predictive of SCD risk[9], much like the findings in the current pediatric study. Separate studies have reported THBS1 over-expression in the hypertrophied right ventricle of a pulmonary hypertension mouse model[31], overexpression in the LV myocardium of a mouse models of cardiac hypertrophy[32,33], and overexpression in patients with LVH secondary to aortic stenosis.[33] A more recent study comparing gene-expression patterns of an *in vitro* human stem-cell-based cardiac hypertrophy model with the gene expression of myocardial biopsies collected from hypertrophic human hearts, confirmed the overexpression of THBS1 in both[34].
Talin I (TLN1), a large dimeric cytoskeletal protein, activates integrins and links it to the actin cytoskeleton. Integrins are key mechanotransducers in cardiomyocytes and therefore intimately involved in the process of cardiac hypertrophy. TLN1 was found to be up-regulated in the costameres of a HCM mouse model, in the myocardium of adult humans with heart failure[35] and our previous work showed increased plasma abundance in patients with HCM.[9] In addition, it has been shown that cardiomyocyte-specific TLN1 deletion in knock-out mice alters physiological and molecular responses of the myocardium to stress, leading to improved cardiac remodelling following pressure overload[35].
The human protein-protein interaction network for our 7 biomarkers (**Figure S1c**), illustrates how the ALDOA, THBS1 and TLN1 clusters share a common interaction with Fibronectin 1 (FN1), which also mediates the interaction with LPA. FN1 has previously been shown to contribute to pathological cardiomyocyte hypertrophy[36].

Description and function of prognostic proteomics biomarkers for HCM
APOL1 (Apolipoprotein L1) may serve as an inflammatory mediator related to autophagy and pyroptosis[37]. Abnormalities in the *APOL1* gene have been implicated in the burden of end-stage kidney disease in persons of African ancestry and more recently it has been linked to increased cardiovascular events and sudden cardiac death in patients receiving maintenance hemodialysis of European ancestry[38].
C5b (Complement 5b), that plays a role in the membrane attack complex (MAC), along with C5a, a potent pro-inflammatory molecule, are the downstream cleavage products of the complement component C5. C5a is essential for the recruitment and activation of inflammatory cells such as granulocytes and it mediates its effect primarily by binding a G-protein coupled receptors. It has previously been shown that the hearts of C5 deficient mice were in a state of distress, conferring a predisposition to heart failure and death in the face of additional injury such as incident infection.[21,39]
IGHE (Immunoglobulin Heavy Constant Epsilon) belongs to a class of immunoglobulins involved in immune response to specific allergens and its role in cardiac disease is still being elucidated. On the one hand, patients with reduced serum IgE levels were at higher risk of sudden cardiac death following acute myocardial infarction due to accelerated clot formation and early appearance of thrombin in their coronary arteries[40]. On the other hand, another study reports that higher IgE levels were associate with adverse cardiac remodelling and cardiac dysfunction[41].
SAA4 (Serum Amyloid A4) is encoded by one of four SAA genes and it is constitutively expressed[42] which is known to be abundantly expressed in healthy mouse and human heart tissue. To date nothing is known about the role of SAA4 in HCM pathophysiology or risk for sudden cardiac death.

Supplemental Tables
Table S1. Genetic status of children with HCM recruited into the study.

No.	Gene Family – Amino Acid Mutation	
Sarcomeric variants		
1	*MYBPC3*	c.1224-5G>A
2	*MYBPC3*	c.1790G>A(p.Arg597Gln)
3	*MYBPC3*	c.1505G>A(p.Arg502Gln)
4	*MYBPC3*	c.1484G>A(p.Arg495Gln)
5	*MYBPC3*	c.2458C>T(p.Arg820Trp)
6	*MYBPC3*	c.1504C>T(p.Arg502Trp)
7	*MYBPC3*	c.1484G>A(p.Arg495Gln)
8	*MYBPC3*	c.772G>A(p.Glu258Lys)
9	*MYBPC3*	c.3330+5G>C
10	*MYBPC3*	c.2373_2374insG(p.Trp792Valfs*41)
11	*MYBPC3*	c.3413G>C(p.Arg1138Pro)
12	*MYBPC3*	c.1504C>T(p.Arg502Trp)
13	*MYBPC3*	c.927-9G>A
14	*MYBPC3*	c.2308G>A(p.Asp770Asn)
15	*MYBPC3*	c.2610delC(p.Ser871Alafs*8)
16	*MYBPC3*	c.927-2A>G
17	*MYBPC3*	c.2054_2067+11del25(p.Lys685fs)
18	*MYBPC3*	c.1624+4A>T
19	*MYBPC3*	c.3190+5G>A
20	*MYBPC3*	c.305delCinsTGAGG(p.Pro102Leufs*12)
21	*MYBPC3*	c.3548T>G(p.Phe1183Cys)
22	*MYBPC3*	c.1504C>T(p.Arg502Trp)
23	*MYBPC3*	c.772G>A(p.Glu258Lys)
24	*MYBPC3*	c.1321G>T(p.Glu441*)
25	*MYH7*	c.739T>C(p.Phe247Leu)
26	*MYH7*	c.1358G>A(p.Arg453His)
27	*MYH7*	c.2462T>G(p.Phe821Cys)
28	*MYH7*	c.2666T>A(p.Leu889His)
29	*MYH7*	c.2779G>A(p.Glu927Lys)
30	*MYH7*	c.2221G>T(p.Gly741Trp)
31	*MYH7*	c.2701G>A(p.Ala901Pro)
32	*MYH7*	c.2156G>A(p.Arg719Gln)
33	*MYH7*	c.1120G>A(p.Glu374Lys)
34	*MYH7*	c.2872G>A(p.Glu258Lys)
35	*MYH7*	c.2087A>G(p.Asn696Ser)
36	*MYH7*	c.1216G>A(p.Val406Met)
37	*MYH7*	c.2791_2793delGAG(p.Glu931del)
38	*MYH7*	c.2791_2793delGAG(p.Glu931del)
39	*MYH7*	c.2770G>A(p.Glu924Lys)
40	*MYH7*	c.2221G>T(p.Gly741Trp)
41	*MYH7*	c.2156G>A(p.Arg719Gln)
42	*MYH7*	c.1357C>T(p.Arg453Cys)
43	*MYH7*	c.2167C>G(p.Arg723Gly)
44	*MYH7*	c.1208G>T(p.Arg403Leu)
45	*MYH7*	c.2551T>C(p.Ser851Pro)
46	*MYH7*	c.1318G>A(p.Val440Met)
47	*MYH7*	c.2539A>G(p.Lys847Glu)
48	*MYH7*	c.2770G>A(p.Glu924Lys)
49	*MYH7*	c.2011C>T(p.Arg671Cys)
50	*MYH7*	c.2221G>T(p.Gly741Trp)
51	*MYH7*	c.2779G>A(p.Glu927Lys)
52	*MYL2*	c.496G>C(p.Asp166His)
53	*TNNT2*	c.517_519del(p.Glu163del)
54	*TNNT2*	Unavailable
55	*TNNI3*	c.616A>C(p.Lys206Gln)
56	*TNNI3*	c.602T>A(p.Met201Lys)
57	*ACTC1*	c.301G>A(p.Glu101Lys)
58	*TPM1*	c.574G>A(p.Glu192Lys)
59	*TPM1*	c.523G>A(p.Asp175Asn)
60	*TPM1*	c.574G>A(p.Glu192Lys)
Syndromic		
61	*AGK*	c.1047-2A>G
62	Carnitine transporter deficiency	
63	*FXN*	
64	*FXN*	
65	*FXN*	
66	*FXN*	
67	*FXN*	
68	*FXN*	
69	*FXN*	
70	*FXN*	
71	*FXN*	

72	*FXN*	
73	*FXN*	
74	*FXN*	
75	*GSD type III*	
76	*GSD type III*	
77	*LAMP2*	c.294G>A(p.Trp98X)
78	*LAMP2*	c.294G>A(p.Trp98X)
79	*MTTI*	m.4300A>G
80	*PTPN11*	c.836A>G(p.Tyr279Cys)
81	*PTPN11*	c.923A>G(p.Asn308Ser)
82	*PTPN11*	c.1528C>G(p.Gln510Glu)
83	*PTPN11*	c.188A>G(p.Tyr 63Cys)
84	*PTPN11*	c.836A>G(p.Tyr279Cys)
85	*PTPN11*	c.1492C>T(p.Arg498Trp)
86	*PTPN11*	c.836A>G(p.Tyr279Cys)
87	*PTPN11*	c.836A>G(p.Tyr279Cys)
88	*PTPN11*	c.236A>G(p.Glu79Arg)
89	*RAF1*	c.770C>T(p.Ser257Leu)
90	*RAF1*	c.1082G>C(p.Gly361Ala)
91	*RAF1*	Not available
92	*RAF1*	Not available
93	*RAF1*	Not available
94	*RAF1*	Not available
95	*RAF1*	Not available
96	*RIT1*	c.295T>A(p.Phe99Ile)
97	*RIT1*	c.244T>C(p.Phe82Leu)
Other		
98	*DES*	c.1216C>T(p.Arg406Trp)
99	*Chromosome 16q22.1 deletion*	
100	*JPH2*	c.494C>T(p.Ser165Phe)
VUS		
101	*ACTN2*	c.2161C>T(p.Arg721Cys)
102	*ACTC1*	c.2072_2074delACA(p.Asn691del)
103	*ACTC1*	c.2072_2074delACA(p.Asn691del)
104	*CASQ2*	c.118A>G(p.Lys40Glu)
105	*FHOD3*	c.1910G>A(p.Arg637Gln)
106	*FHOD3*	c.1943T>C(p.Ile648Thr)
107	*HRAS*	c.182A>C(p.Gln61Pro)
108	*JPH2*	c.479G>C(p.Arg160Pro)
109	*MYH7*	c.5088G>C(p.Glu169Asp)
110	*MYH7*	c.2479T>C(p.Trp827Arg)
111	*MYH7*	c.1012G>C(p.Val338Leu)
112	*MYH7*	c.635G>A(p.Gly212Asp)
113	*MYL3*	c.452C>A(p.Ala151Asp)
114	*MYOM1*	c.1_929del
115	*PLN*	c.63_64dup(p.Gln22fs)
116	*RAF1*	c.1553G>A(p.Arg518Gln)
117	*RAF1*	c.671T>G(p.Met224Arg)
118	*SOS1*	c.3286T>A(p.Ser1096Thr)
119	*SOS1*	c.3355A>G(p.Thr1119Ala)
120	*TPM1*	c.755G>C(p.Ser252Thr)
121	*TPM1*	c.115-10C>G

Remaining patients not listed here were found to be gene elusive or were not tested.

Table S2. Differentially abundant peptides between HCM and controls in the HCM plasma profiling experiments.

Peptides	Control	Overt HCM	*p*-Value
**C3**	914869 ± 92031	1079910 ± 107436	**0.014**
**C4BPB**	18654 ± 3999	32655 ± 11632	**0.014**
**CFD**	7822 ± 2229	13756 ± 3734	**0.012**
**ECM1**	53448 ± 9720	77559 ± 14052	**0.019**
**HPX**	635824 ± 124913	890455 ± 85700	**0.007**

Peptides without statistically significant differences are not shown.
Normalized peptide abundances are reported as mean ± SD.
C3, Complement C3; C4BPB, C4b-binding protein alpha chain; CFD, Complement factor D; ECM1, Extracellular matrix protein 1; HPX, Hemopexin.

Table S3. Optimal cut-offs for biomarkers considering single analytes as opposed to multi-marker panels.

Panels	Gene	Name	Single Analyte Cut-off [Accuracy, Sensitivity, Specificity | AUC]
Diagnostic	ALDOA	Aldolase Fructose-Bisphosphate A	≥0.11 pmol/mL [70%, 74%, 54% | 0.67]
	C3	C3a anaphylatoxin	≥15.5 pmol/mL [59%, 57%, 64% | 0.61]
	LPA	Lipoprotein, Lp(A)	≥6.95 pmol/mL [58%, 55%, 66% | 0.60]
	PFN1	Profilin-1	≥0.44 pmol/mL [55%, 50%, 70% | 0.59]
	PYGB	Glycogen phosphorylase, brain form	≥1.90 pmol/mL [61%, 61%, 60% | 0.61]
	THBS1	Thrombospondin 1	≥5.85 pmol/mL [68%, 77%, 42% | 0.60]
	TLN1	Talin-1	≥0.03 pmol/mL [73%, 85%, 36% | 0.63]
Prognostic	APOL1	Apolipoprotein L, 1	≤7.37 pmol/mL [65%, 66%, 62% | 0.60]
	C5b	Complement C5 beta chain	≤3.71 pmol/mL [55%, 42%, 82% | 0.63]
	IGHE	Immunoglobulin heavy constant epsilon	≤0.14 pmol/mL [58%, 43%, 87% | 0.64]
	SAA4	Serum amyloid A-4 protein	≤6.60 pmol.mL [55%, 42%, 82% | 0.62]

Accuracy defined as = True Positive + True Negative / True Positive + True Negative + False Positive + False Negative.
AUC = area under the curve.

Table S4. Confusion matrix for the SVM analysis of training dataset.

	PREDICTED		
**ACTUAL**	**HCM**	**Control**	*Total*
**HCM**	104	0	*104*
**Control**	0	96	*96*
Total	*104*	*96*	*200*

Table S5. Confusion matrix for the SVM analysis of validation dataset.

	PREDICTED		
**ACTUAL**	**HCM**	**Control**	*Total*
**HCM**	33	7	*40*
**Control**	11	49	*60*
Total	*44*	*56*	*100*

Supplemental Figures

Figure S1. Ranked pathway analysis for abundant proteins in HCM and human interactome.

(**a**) Ranked pathway analysis using the 59-protein multi-marker assay. Red numbers in squared brackets refer to the number of enriched genes per pathway relative to the total number of assayed genes (59). Size of dots represents number of enriched genes within the assay and intensity of colours represents the P-value associated with enrichment analysis. (**b**) Network analyses for ranked pathway analysis (showing top 10 terms only) between children with HCM and controls, where high abundance proteins are coloured green and low abundance proteins are coloured red. Node size of the pathway term reflects the number of input proteins implicated in the pathway. (**c**) Human protein interaction network for the 7 key diagnostic biomarkers in childhood-onset HCM (highlighted in yellow) excluding non-human nodes and edges.

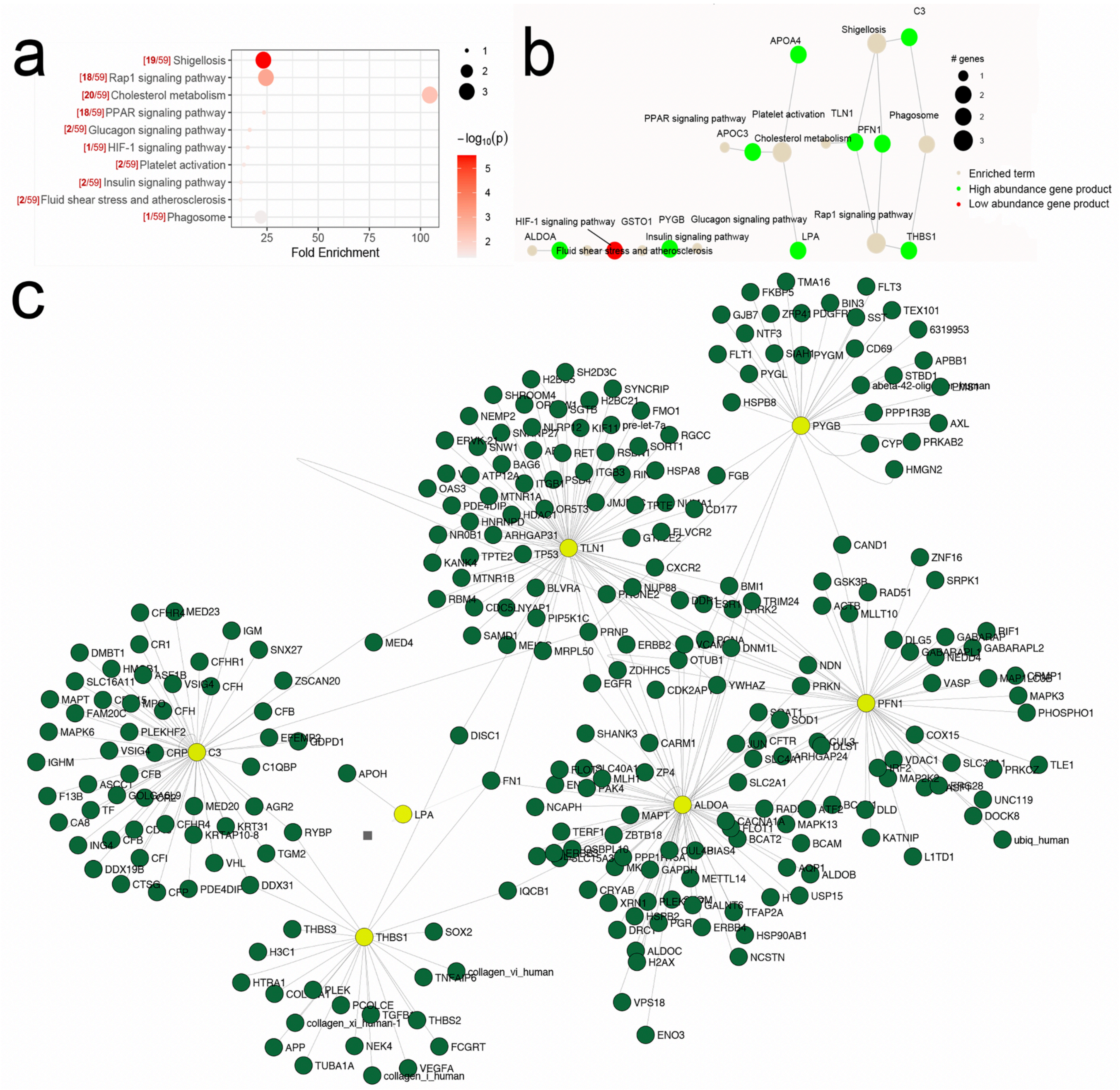

Figure S2. Additional survival curves for arrhythmic endpoint that did not reach statistical significance.
Survival curves for single analytes, APOL1 and SAA4 and the arrhythmic endpoint.
HR, hazard ratio.

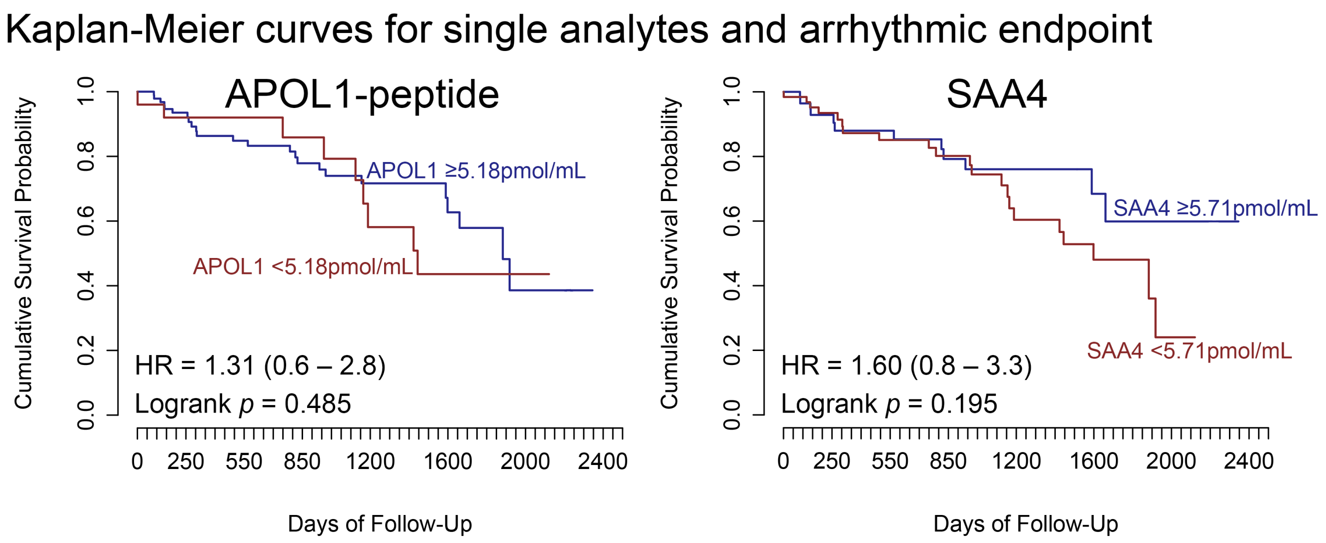

Supplement: Supplementary file 1 [file hcg-17-e004448-s001.pdf]
